# Serendipitous Discovery of a Guanine-rich DNA Molecule with a Highly Stable Structure in Urea

**DOI:** 10.1038/s41598-018-20248-w

**Published:** 2018-01-31

**Authors:** Wenqing Zhang, Meng Liu, Christine Lee, Bruno J. Salena, Yingfu Li

**Affiliations:** 1 Department of Biochemistry and Biomedical Sciences, McMaster University, 1280 Main St W, Hamilton, ON L8S 4K1 Canada; 2 Department of Pathology and Molecular Medicine, McMaster University, 1280 Main St W, Hamilton, L8S 4K1 ON Canada; 3 Department of Medicine, McMaster University, 1280 Main St W, Hamilton, ON L8S 4K1 Canada

## Abstract

We have made an accidental discovery of an unusual, single-stranded, guanine-rich DNA molecule that is capable of adopting a folded structure in 7 M urea (7MU) known to denature nucleic acid structures. The folding of this molecule requires Na^+^ and Mg^2+^ and the folded structure remains stable when subjected to denaturing (7MU) polyacrylamide gel electrophoresis. Results from sequence mutagenesis, DNA methylation, and circular dichroism spectroscopy studies suggest that this molecule adopts an intramolecular guanine-quadruplex structure with 5 layers of guanine tetrads. Our finding indicates that DNA has the ability to create extremely stable structural folds despite its limited chemical repertoire, making it possible to develop DNA-based systems for unconventional applications.

## Introduction

DNA is a rather simple polymer from the chemical functionality perspective, especially when compared to proteins. This is because DNA is built with four chemically similar nucleobases distributed over a backbone of sugar-phosphodiesters. And yet, many man-made DNA molecules have been produced, through the technique of *in vitro* selection^1,2^, to recognize a broad range of molecular targets or catalyze various chemical transformations^3–20^. However, the majority of functional DNA molecules have been derived to perform tasks under conditions conducive to nucleic acid structure folding, such as neutral pH and amiable temperature. Previous examples of functional DNA molecules that are active under challenging reaction conditions include RNA-cleaving DNAzymes working at high acidity^21–24^ or high temperature^25^, although these DNAzymes exhibit much reduced catalytic efficiency than their counterparts derived to work at neutral pH and normal temperature^25^. Nevertheless, it is largely unknown if single-stranded DNA can create intricate structures under structure-disrupting conditions.

Urea at high concentrations represents a disruptive condition for nucleic acid structures^26,27^. Because urea can act as hydrogen-bond donor and acceptor, it can easily denature structures of nucleic acids. In fact, 6–8 M urea is the key component for denaturing polyacrylamide gel electrophoresis (dPAGE) widely used to separate DNA oligonucleotides by size^28,29^. In this technique, DNA molecules are completely denatured and migrate as unfolded linear polymers through the polyacrylamide gel matrix. To our great surprise, we came across a DNA molecule that can form a folded structure in 7 M urea (7MU), which remains stable during the process of 7MU-dPAGE.

**Figure 1:**
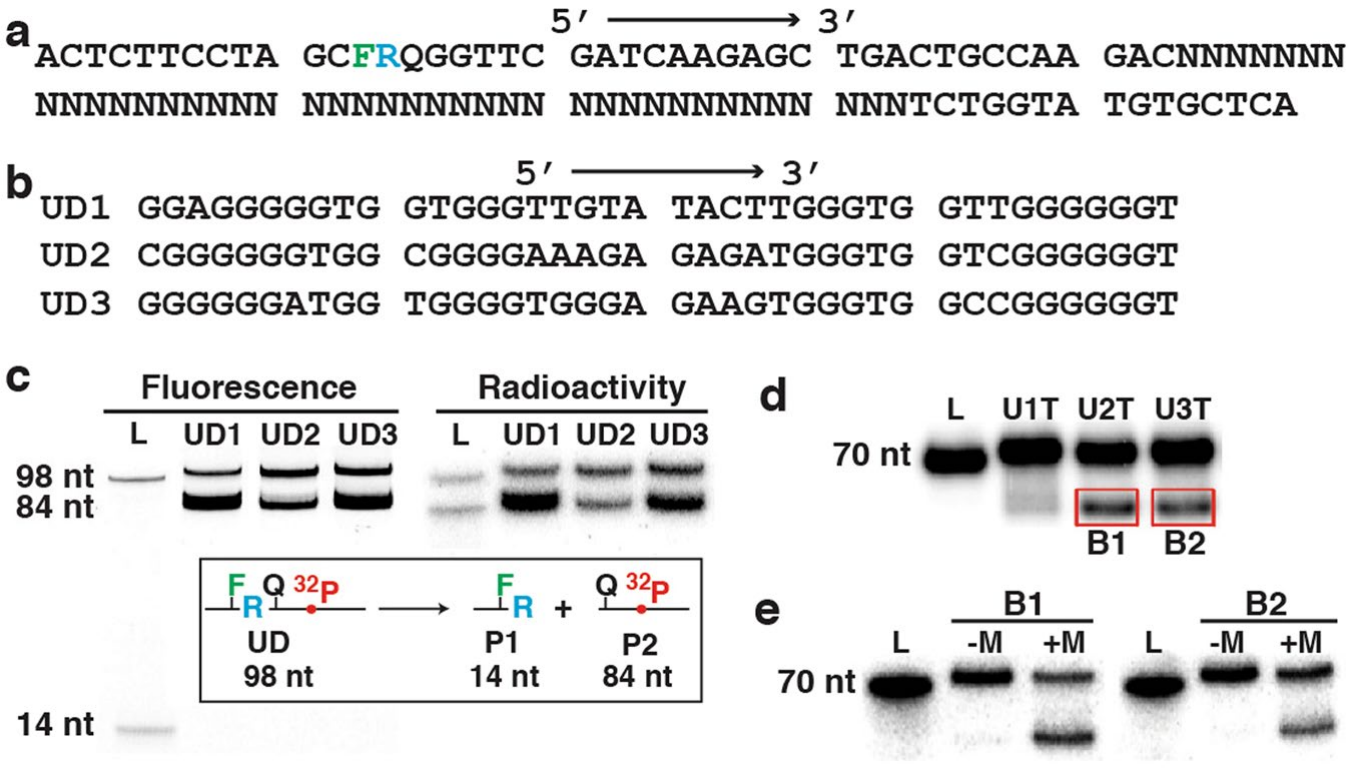
Discovery of DNA sequences with urea-resistant structures. (**a**) The sequence of the DNA library used for *in vitro* selection. R: adenosine ribonucleotide; F: fluorescein-labeled dT; Q: dabcyl-labeled dT; N: mixture of ACGT (25% each). (**b**) The random-sequence regions of UD1, UD2 and UD3. (**c**) Fluorimage and phosphorimage of a 7MU-dPAGE gel conducted to analyze the cleavage reaction of UD1–3. Insert: The cleavage reaction with 14-nt P1 and 84-nt P2 as the cleavage product. L: DNA ladder lane. (**d**) 7MU-dPAGE analysis of U1T, U2T and U3T (truncated UD1, UD2 and UD3 with the removal of the first 28 nucleotides). (**e**) 7MU-dPAGE analysis of the boxed DNA bands from panel d. + M and -M: with and without 50 mM NaCl and 5 mM MgCl_2_, respectively. Cropped gel images were used in panels (**c**–**e**) (uncropped gel images are provided in Fig. S6).

The original goal of this study was to use *in vitro* selection technique to isolate RNA-cleaving DNAzymes to function under the condition of 20 mM Tris-HCl, pH 8.5, 50 mM NaCl, and 10 mM MgCl_2_ (denoted 1× Selection Buffer, or 1× SB), from a random-sequence synthetic DNA pool. The DNA pool was made of ~10^14^ 98-nt DNA molecules (nt: nucleotide) with 43-nt constant sequence at the 5′-end, followed by 40-nt random sequence and 15-nt constant sequence (Fig. 1a). Note that the 5′-sequence contains an adenosine ribonucleotide at 14^th^ position (R in Fig. 1a) as the cleavage site, which is surrounded by a pair of thymine deoxyribonucleotides modified with a fluorescein (F) and the dabcyl quencher (Q)—a design intended for making DNAzymes for biosensing application^30,31^. The selection strategy, depicted in Fig. S1, is straightforward: RNA-cleaving DNA sequences produce two products, 14-nt P1 and 84-nt P2. P2 is the desired product because it contains the catalytic DNA sequence. Because of large differences in size, P2 can easily be separated from both P1 and the uncleaved molecules using 7MU-dPAGE. Purified P2 is first multiplied via DNA amplification and then subjected to the next cycle of cleavage, product separation and DNA amplification. This procedure is repeated until a strong cleavage activity is observed.

The described selection strategy has been successfully used to derive RNA-cleaving DNAzymes in our previous studies^21,30–33^. As expected, a strong P2 DNA band on 7MU-dPAGE gel was observed after 16 cycles of selection. The DNA pool was then subjected to cloning and sequencing. 33 sequences were obtained, which can be grouped into 3 sequence classes (Table S1). A representative sequence from each class was chosen for further characterization (Fig. 1b). These sequences are named UD1, UD2, and UD3.

The first evidence suggesting that these DNA molecules were not RNA-cleaving DNAzymes came from the experiment depicted in Fig. 1c. For this experiment, each UD was made to have a ^32^P-labeled phosphodiester linkage between the 28^th^ and 29^th^ nucleotides in addition to the fluorescein label at the 13^th^ position. Therefore, the RNA cleavage reaction should produce a fluorescent P1 and a radioactive P2, as illustrated in the inserted reaction scheme in Fig. 1c. The uncleaved UD should be detectable by both fluorimaging (gel image on the left) and radioimaging (image on the right), while P1 and P2 should be detectable only by fluorimaging and radioimaging, respectively. However, P1 was not detected at all whereas P2-like DNA bands appeared in both images. These observations strongly suggest that we obtained novel DNA sequences that are able to fold into stable structures in 7MU. The folded DNA molecules are more compact than unfolded DNA sequences, and thus migrate faster on 7MU-dPAGE. These molecules were selected simply because the folded structure (albeit 98-nt in length) has a gel mobility that is similar to the linear 84-nt DNA molecule used as the gel excision marker.

To provide additional evidence, we performed an experiment using altered UD1–3 (named U1T, U2T and U3T; Fig. 1d) where the first 28 nucleotides (including the RNA cleavage site) were removed. A faster migrating DNA band appeared for each shortened DNA, particularly for U2T and U3T.

The folded DNA band from U2T and U3T, labeled B1 and B2, were excised from the gel, eluded, denatured by heat, and refolded under the *in vitro* selection condition. When they were subjected to dPAGE analysis, two DNA bands were observed again. This experiment further verified that UD1, UD2 and UD3 are not RNA-cleaving DNAzymes. More importantly, the appearance of the fast-migrating DNA band was dependent on the presence of 50 mM NaCl and 5 mM MgCl_2_ (“+M” lanes, Fig. 1e).

We realized by this time that the experimental procedure used for *in vitro* selection in this study had two key deviations from the protocol previously used by us for isolating RNA-cleaving DNAzymes. Our standard practice has a step of quenching the cleavage reaction by adding sufficient amount of EDTA to chelate divalent metal ions, followed by DNA recovery using ethanol precipitation. The DNA is then dissolved in 1× dPAGE gel loading buffer containing 7MU (1× dGLB), heated at 90 °C for 5 min and cooled at room temperature for 15 min. The mixture is then subjected to 7MU-dPAGE to purify the cleavage product. However, in the current study, the cleavage reaction mixture was directly combined with 2× dGLB, followed by heating, cooling and 7MU-dPAGE purification as usual. In other words, the altered protocol omitted EDTA-chelation and ethanol-precipitation steps. These omissions resulted in a DNA mixture containing 50 mM NaCl and 5 mM MgCl_2_ in addition to 7MU prior to gel loading. We believe that the presence of metal ions in the loading mixture provided a chance for UD sequences to fold into a tight structure in the presence of 7MU and get selected via isolation on 7MU-dPAGE.

**Figure 2:**
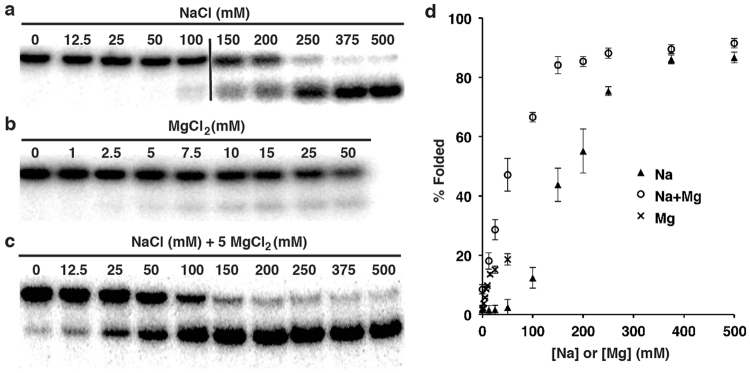
Metal ion requirements for the folding of U2T. (**a**) Effect by Na^+^ (in the absence of Mg^2+^). (**b**) Effect by Mg^2+^ (in the absence of Na^+^). (**c**) Effect by Mg^2+^ and Na^+^. Each reaction mixture contained 44.5 mM Tris-borate, 10 mM HEPES, 7 M urea, and the specified metal ions. (**d**) % Folded structure vs. NaCl concentration. The mixture was heated at 90 °C for 5 min and left standing at room temperature for 15 min before loading. Cropped gel images were used for panel (**a**–**c**) (uncropped gel images are provided in Fig. S6). In addition, for panel (**a**), two different gels, separated by the black dividing line, were grouped.

To probe into this hypothesis, we carried out an experiment to examine the concentration effect of Na^+^ and Mg^2+^ on the folding of U2T. Sodium itself at high concentrations (375 mM or above; Fig. 2a,d) can induce a high level (~85%) of structural formation of U2T. Magnesium was less effective: only ~20% U2T were folded when 100 mM MgCl_2_ was provided (Fig. 2b,d). However, a synergistic effect was seen when Na^+^ and Mg^2+^ were used simultaneously: in the presence of 5 MgCl_2_, ~85% U2T adopted folded structure when the sodium concentration reached 150 mM. K^+^, Li^+^ and NH_4_^+^ were also found to support the structural formation (Fig. S2), and the effectiveness of the tested monovalent cations follows the order of Na^+^ > K^+^ > NH_4_^+^ > Li^+^.

**Figure 3:**
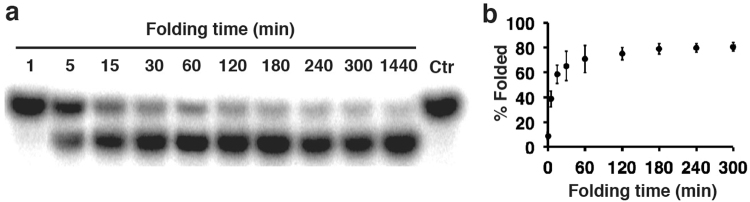
Time for folding. (**a**) 7MU-dPAGE analysis. A cropped gel image was used (the uncropped gel image is provided in Fig. S6). (**b**) % Folded structure vs. folding time. U2T was heated at 90 °C for 5 min and then incubated at room temperature for the specified time before 7MU-dPAGE analysis. Ctr: the reaction mixture was immediately loaded following heat denaturation. All the reaction mixtures contained 7MU, 5 mM MgCl_2_, and 150 NaCl.

We next investigated the time for folding. U2T samples containing 150 mM NaCl, 5 mM MgCl_2_, and 7MU were heated at 90 °C for 5 min and then incubated at room temperature for times specified in Fig. 3a prior to dPAGE analysis. Note that each folding reaction was started at a different starting time so that all the reactions reached the same endpoint for gel loading. The graph presented in Fig. 3b indicates that ~40% U2T adopted the folded structure in 5 min, 70% in 60 min, and additional 10% molecules were able to fold in the next 3 h. The last lane was a control where a sample was loaded immediately following the heating step, and the lack of fast-migrating DNA band in this lane indicates that U2T was unable to fold within the gel, most probably due to the absence of metal ions in the gel. The folded structure, however, remained stable within the gel.

**Figure 4:**
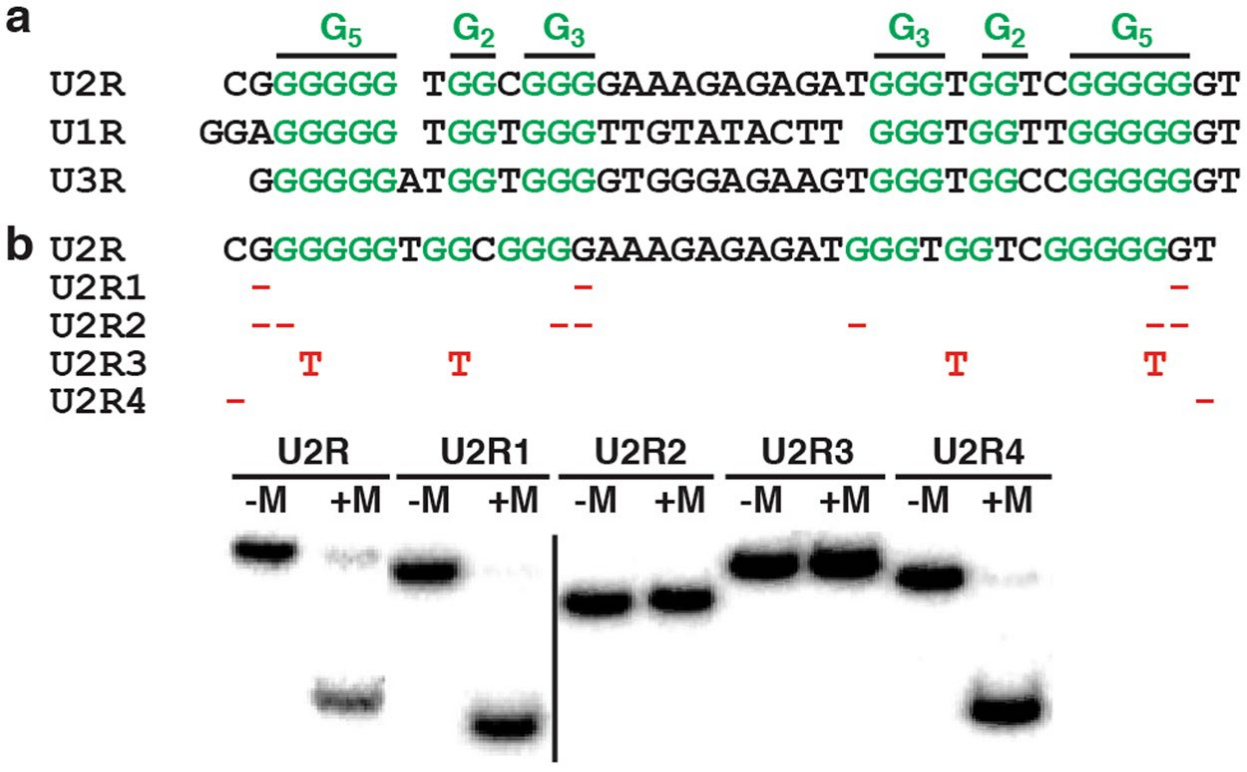
Testing variant sequences of U2R. (**a**) Sequence alignment of U2R, U1R and U3R. Guanines with significant methylation interference are shown in green. Only altered nucleotides are shown. -:nucleotide deleted; red T: guanine mutated to thymine. (**b**) Analysis of folding property of U2R mutants in 7MU with (+) and without (−) of 5 mM MgCl_2_, and 150 NaCl. Two different gels, separated by the black dividing line, were grouped. In addition, cropped gel images were used (uncropped gel images are provided in Fig. S6).

Sequence truncation experiment provided in Fig. S3 indicates that nucleotides outside of the random region can be completely removed. The 40-nt UD2 is named U2R. It is highly rich in guanine (G; 65%) and such sequences tend to form G-quadruplex structures comprising stacks of G-quartets, planar arrangement of four guanines bonded with 8 hydrogen bonds^34–40^. Footprinting experiment with dimethyl sulfate (DMS) was then performed with U2T and the data is provided in Fig. S4. This technique can detect the sensitivity of N7 atoms of guanines in G-quartets to methylation (so called “methylation interference”). It was found that 20 of 26 guanines exhibited significant methylation interference (green G residues in Fig. 4a), indicating that these guanine bases play important roles in UD2’s structural folding. Interestingly, these guanines are conserved in U1R and U3R (Fig. 4a) and distributed into six G-stretches in the following arrangement: G_5_N_1–2_G_2_N_1_G_3_N_10–11_G_3_N_1_G_2_N_2_G_5_ (N stands for A, C or T). This finding suggests that these 3 selected DNA molecules are sequence relatives.

The folding property of several U2R mutants (Fig. 4b) were examined next. Removing 3 internal, non-conserved guanine nucleotides (U2R1) had no effect on folding. However, deleting (U2R2) or mutating (U2R3) conserved guanine nucleotides was totally disruptive. As expected, the 5′ C and 3′ T (U2R4) nucleotides had no effect.

**Figure 5:**
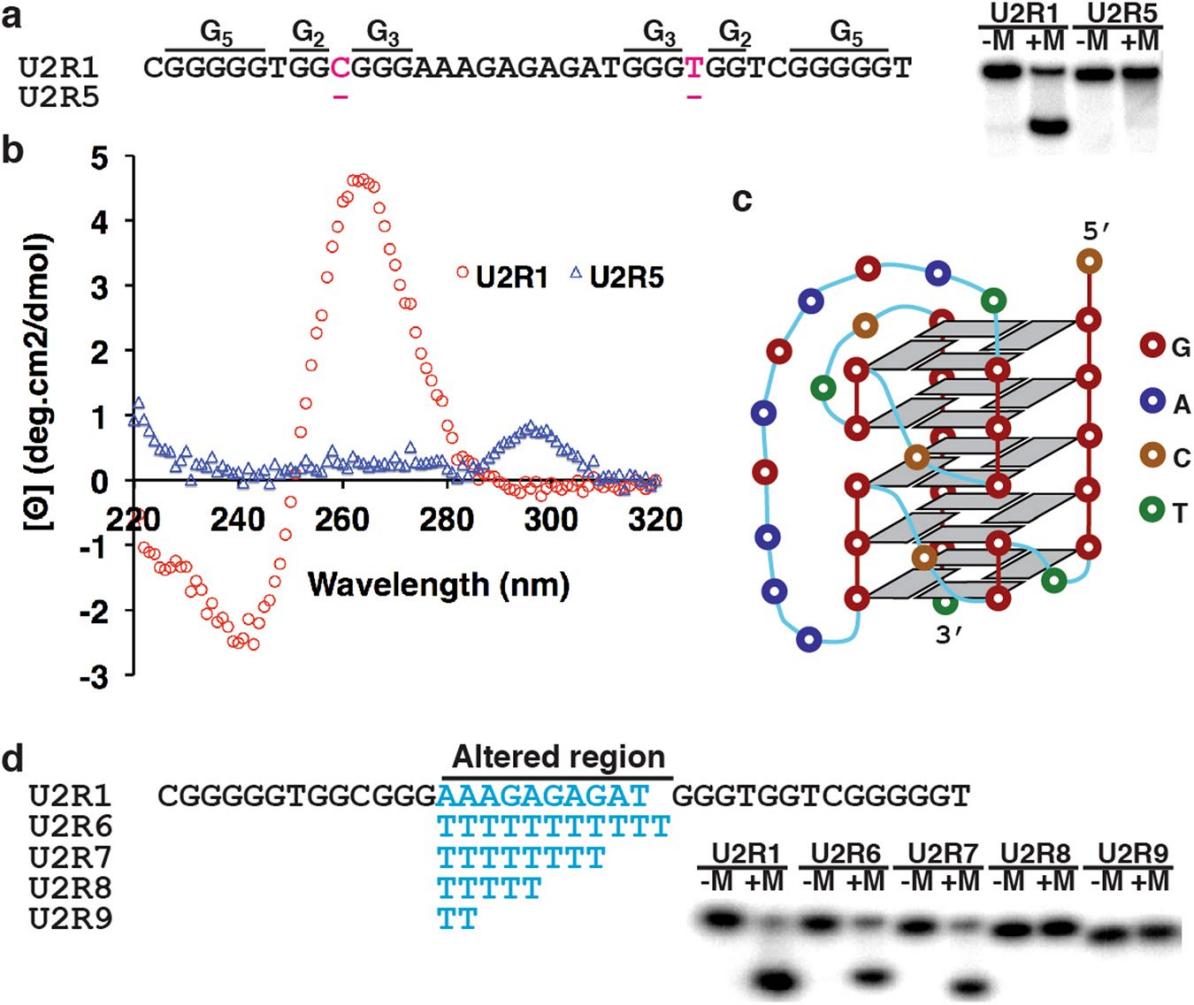
A structure model for U2R1. (**a**) Analysis of folding property of U2R1 and its mutant U2R5 in the presence of 7MU. (**b**) Circular dichroism spectra obtained with U2R1 and U2R5 in 7MU, 150 mM NaCl and 5 mM MgCl_2_. (**c**) Proposed structure model. (**d**) Analysis of folding property of U2R1 mutants altered at the long linking loop. +M and −M in panels (**a**,**d**): with and without 150 mM NaCl and 5 mM MgCl_2_. For panels (**a**,**d**), cropped gel images were used (uncropped gel images are provided in Fig. S6).

The conserved sequence motif has two G_5_ elements and two G_2_+G_3_ elements each with a pyrimidine nucleotide insertion (pink C and T, Fig. 5a). This observation prompted us to examine whether these two nucleotides are essential to the structural folding, particularly considering their removal would produce two additional G_5_ elements to allow the DNA sequence to form a quadruplex structure with 5 layers of G-tetrads. Surprisingly, these two nucleotides are indispensable to the structural folding (U2R5, Fig. 5a)—this point will be further discussed below.

Circular dichroism spectroscopy (CD) has been widely used to examine guanine quadruplex structures, particularly for identifying different G-quadruplex folds^36,41–45^. The CD spectrum of U2R1 in the presence of urea (unfilled circles, Fig. 5b) contains a positive peak at 260 nm and a negative peak at 240 nm, both of which were not seen in the spectrum of U2R5 (unfilled triangles). The CD spectrum of U2R1 is indicative of a parallel G-quadruplex fold extensively reported in the literature for other G-quadruplex forming DNA and RNA sequences, strongly suggesting that U2R1 has G-quadruplex made of exclusively parallel DNA strands^46–48^. The parallel chain directionality of U2R1 was further tested with protoporphyrin (PPIX), which has been shown to selectively bind to parallel G-quadruplexes and produce fluorescence enhancement^49^. Indeed, strong fluorescence increases were observed when PPIX was incubated with U2R1 both in the absence and presence of 7MU (Fig. S5a).

Based on the above information, we propose a structural model, which is depicted in Fig. 5c. The proposed structure consists of two continuous stacks of tetrads (top and bottom two-tiers) sandwiching a middle stack with two backbone openings. The model not only places all 20 conserved guanines into 5-layer G-quartets, but the nature of all parallel strands is also consistent with the CD data (Fig. 5b) and the results from the PPIX binding experiment (Fig. S5a). Furthermore, the model explains the indispensability of the aforementioned pyrimidine nucleotides because they are needed for creating all-parallel strand orientations. The structure also has a long linking loop (10 nt) connecting two distal guanines located on the top and bottom tetrads, respectively. We found that nucleotide identities are not important as they can be changed to thymidines (U2R7, Fig. 5d). However, the number of nucleotides in this loop has pronounced impact on formation of the urea-resistant structure: while 8 and more nucleotides (U2R6, 11 thymidines; U2R7, 7 thymidines) support the structure formation, shortened loops (5 nt in U2R8 and 2 nt in U2R9) are completely ineffective. These findings are consistent with the proposed structural model.

We examined the thermal stability of U2R1 by measuring their melting points (Tm values) in the absence and presence of 7MU and the data are shown in Fig. S5b. U2R1 has a melting point of 68 °C and 59 °C in the absence and presence of 7MU, and more importantly, its Tm decreases only by 9 °C in the presence of 7MU. These results are consistent with a highly stable structure associated with U2R1 even in the presence of 7MU.

Finally, we tested whether the RNA version of U2R1 (named RNA-U2R1) could fold into a similar urea-resistant structure. Interestingly, RNA-U2R1 behaved very differently from U2R1 as it did not produce a defined fast-migrating band on the 7MU-dPAGE (Fig. S5c). This particular observation suggests that the stable structure adopted in 7MU by U2R1 does not straightforwardly translate into a similar stable structure by the RNA with the same sequence. However, we believe RNA molecules with similar properties do exist and can be isolated from random-sequence RNA pools.

In summary, we have accidently discovered a remarkable DNA molecule that can adopt a folded structure in the presence of 7 M urea, which is known to be a strong denaturant for nucleic acid structures. This DNA molecule is highly rich in guanine and several lines of evidence suggest that this molecule forms an intricate 5-tiered guanine-quadruplex with all-parallel strand orientations. To our knowledge, such a structural arrangement has never been described before. The formation of the structure is contingent on the presence of metal ions. Overall, our work demonstrates that DNA, despite its chemically simplicity, is able to create highly stable structural folds in a structurally disruptive environment. This finding opens up the possibility of developing DNA-based systems for unconventional applications. For example, it is conceivable that DNA aptamers or ligand-responsive DNAzymes for biosensing applications can be isolated to function under denaturing conditions designed to overcome issues such as non-specific binding or degradation by nucleases in biological samples. Our finding may also have implications for origin of life. It has been hypothesized that life on Earth may have emerged from an “RNA world” where RNA played the dual roles of being the hereditary material and carrying enzymatic reactions^50–53^. It is possible that the early RNA catalysts may have to deal with structure-disrupting conditions imposed by urea-like denaturants. Furthermore, these unsupportive conditions might also facilitate the replication of nucleic acid enzymes as they can help denature the replicated duplexes at elevated temperature and allow the regeneration of functional enzymes under denaturing conditions.

## Methods

### Enzymes, chemicals, and other materials

T4 DNA ligase and T4 polynucleotide kinase (PNK) were purchased from Thermo Scientific (Ottawa, ON, Canada). [γ-^32^P]dATP was obtained from Perkin Elmer (Woodbridge, ON, Canada). Urea (ultrapure) and 40% polyacrylamide solution (29:1) were acquired from BioShop Canada (Burlington, ON, Canada). The water used was purified via Milli-Q Synthesis A10 water purifier. All other chemicals were purchased from Sigma-Aldrich (Oakville, ON, Canada). 10× TBE (Tris-borate EDTA) (1 L) was made of 108 g Tris-base (0.89 M), 55 g boric acid (0.89 M), 20 mL of 0.5 M ethylenediaminetetraacetic acid (EDTA; pH 8.0; 10 mM). 2× native gel-loading buffer (per 100 mL) was made with 20 g sucrose, 10 mL of 10× TBE, 1 mL of 10% (w/v) SDS (sodium dodecyl sulphate), 25 mg bromophenol blue, and 25 mg xylene cyanol FF. 2× denaturing gel-loading buffer was made with the same recipe, with the addition of 84.1 g urea (14 M). Note that the concentration of Tris-boric acid in the 2× gel-loading buffer was 89 mM. 10% denaturing polyacrylamide gel stock was made of 250 mL of 40% polyacrylamide solution (29:1), 100 mL of 10× TBE, and 425 g urea.

### Synthesis and purification of oligonucleotides

DNA and RNA oligonucleotides were purchased from Integrated DNA Technologies (Coralville, IA, USA), prepared using automated synthesis using standard phosphoramidite chemistry. Each oligonucleotide was purified using 10% denaturing polyacrylamide gel electrophoresis (dPAGE) containing 7 M urea. Note that for the purification of urea-resistant DNA molecules featured in this study, DNA samples in 1× denaturing gel loading buffer at 90 °C for 5 min and then immediately loaded onto dPAGE gel. This procedure was adopted to minimize the structure formation.

### ^32^P-labelling of oligonucleotides

1 μL of [γ-^32^P]ATP (10 μCi) was used to label ~150 pmol of DNA or RNA in 50 μL of 1× PNK buffer A containing 5 units of PNK. After incubation at 37 °C for 30 min, PNK was inactivated by heating at 90 °C for 5 min. The phosphorylated DNA was first concentrated by ethanol precipitation and then purified by 10% dPAGE purification.

### *In vitro* selection procedures

*In vitro* selection was conducted using a similar protocol that we previously described^54^. The *in vitro* selection schematic and all the DNA sequences are provided in Fig. S1. 1000 pmol of DL1 were used as the initial library. The DNA molecules in this pool were first labeled with ^32^P at the 5′-end in the presence of 10 units (U) of T4 PNK, 10 µCi of [γ-^32^P]ATP, and 1× T4 PNK buffer A (using the 10× buffer supplied by the vendor) for 20 min at 37 °C in a reaction volume of 100 µL. This was followed by the addition of non-radioactive ATP to a final concentration of 1 mM and further incubation at 37 °C to ensure complete phosphorylation. The reaction was stopped by heating at 90 °C for 5 min. Upon cooling to room temperature (~23 °C), 1000 pmol of S1 and 1000 pmol of T1 were added. The reaction mixture was then heated to 90 °C for 1 min and cooled to RT. 10 units of T4 DNA ligase and 25 µL of 10× T4 DNA ligase buffer (supplied by the vendor) were added to the reaction mixture (total reaction volume: 250 µL). The ligation reaction was carried out at room temperature for 2 h. The DNA in the mixture was precipitated by ethanol and the ligated product was purified by 10% dPAGE^54^.

The purified DNA above was suspended in 25 µL of H_2_O, heated to 90 °C for 30 s and cooled to room temperature over 15 min. The cleavage reaction was initiated by the addition of 25 µL of 2× selection buffer (40 mM Tris-HCl, pH 8.5, 100 mM NaCl, and 20 mM MgCl_2_), and the reaction mixture was incubated at room temperature for 1 h. The reaction solution was then mixed with 50 µL of 2× denaturing gel loading buffer, which was heated at 90 °C for 5 min and cooled to RT for 15 min^54^. Note that the metal ion concentrations were: [Na^+^] = 25 mM and [Mg^2+^] = 5 mM; the buffering agent Tris concentration were 32.2 mM [(40 mM from 2× SB + 89 mM from 2× dGLB)/4]. This mixture was then subjected for 10% dPAGE purification. A DNA marker that has the identical size to the cleavage product was used to guide the excision of the desired DNA band.

The isolated DNA above was amplified by polymerase chain reaction (PCR) in a volume of 50 µL containing 1× PCR buffer (supplied by the vendor as the 10× buffer), 0.2 mM each of the standard dNTPs, 1.25 U of Tth DNA polymerase, 0.5 µM FP1 (forward primer) and 0.5 µM RP1 (reverse primer). Twelve thermocycles were carried out with the following parameters: 94 °C, 30 s (2 min for the first cycle); 52 °C, 40 s; 72 °C, 45 s. A 1/100-fold dilution of the first PCR product was used for the second PCR using the same condition described above with the exception that RP2 was used instead of RP1. Another PCR2 was performed for internal labeling with ^32^P. This was achieved by following the PCR2 protocol except that 10 µCi of [α-^32^P]dGTP and 0.02 mM non-radioactive dGTP were used to substitute 0.2 mM dGTP. The non-radiolabeled and ^32^P-labeled PCR solutions were combined and the DNA in the mixture was precipitated by ethanol. The desired DNA molecules were purified by 10% dPAGE^54^.

The purified DNA was used to carry out the second selection using the same procedure. 16 selection rounds were conducted. The cleavage product from round 16 was amplified, cloned and sequenced using a protocol we published previously^54,55^. 33 DNA sequences were revealed, which are provided in Table S1.

### General procedure for dPAGE-based folding analysis of urea-resistant DNA molecules

The folding buffer (4× FB) contained 40 mM HEPES, pH 7.5, 600 mM NaCl, and 20 mM MgCl_2_. The general procedure was as follows: a DNA sequence of interest (radioactively labeled; 5 pmol in 5 µL) was first mixed with 5 µL of 4× FB, and then combined with 10 µL of 2× dGLB. The final concentration for each component in the mixture was: [DNA] = 12.5 nM, [Na^+^] = 150 mM, [Mg^+^] = 5 mM, [HEPES] = 10 mM, [Tris-boric acid] = 44.5 mM and [urea] = 7 M. The final volume was 20 µL and the final pH was 8.4 (directly measured). This mixture was heated to 90 °C for 5 min, incubated at room temperature (RT) for 15 min, and then loaded onto a 10% dPAGE gel, which contained 10% polyacrylamide, 7 M urea, 89 mM Tris-boric acid and 1 mM EDTA. The unfolded and folded DNA bands separated by dPAGE gel was imaged with a Typhoon Trio + Imager (GE Healthcare) and the radioactivity of each DNA band was quantified with ImageQuant software (Molecular Dynamics). The percentage of folded structure was then calculated using Microsoft Excel^54^.

### Data availability

All data generated or analyzed during this study are included in this published article and its Supplementary Information.

## Electronic supplementary material

Supplementary Information
