# Supplementary material for: Serendipitous Discovery of a Guanine-rich DNA Molecule with a Highly Stable Structure in Urea
Source: Sci Rep. 2018 Jan 31;8:1935. doi: 10.1038/s41598-018-20248-w (PMC5792554; doi:10.1038/s41598-018-20248-w)
Supplement: Supplementary file 1 — Supplementary Information [file 41598_2018_20248_MOESM1_ESM.pdf]

Serendipitous Discovery of a Guanine-rich DNA Molecule with a Highly Stable Structure in Urea

Wenqing Zhang[1], Meng Liu[1], Christine Lee[2], Bruno J. Salena[3] and Yingfu Li[1]*

[1]Department of Biochemistry and Biomedical Sciences, [2]Department of Pathology and Molecular Medicine, [3]Department of Medicine, McMaster University, 1280 Main St. W., Hamilton, ON L8S 4K1, Canada

*Email: liying@mcmaster.ca

SUPPLEMENTARY METHODS

Experimental details for Figure 1. Panel c: 150 pmol of synthetic oligonucleotides, UD1, UD2 and UD3 were phosphorylated with 10 µCi of [γ-^{32}P]ATP by PNK. The reactions were incubated at 37°C for 15 min, followed by adding 100 nmol of nonradioactive ATP and another 20 min of incubation. They were then ligated to 150 pmol S1 in the presence of 150 pmol T1 and 5 units of T4 ligase. Ligated products were then purified by dPAGE. 5 pmol of full-length UD1, UD2 and UD3 for were used for the experiment. For each DNA, 5 pmol in 5 µL were mixed with 5 µL of 2× SB, and then incubated at RT for 15 min. 10 µL of 2× dGLB were then introduced. The resultant mixture was heated to 90°C and cooled at RT for 15 min. 10 µL (out of 20 µL) were taken for dPAGE analysis. Panel d: 5 pmol of U1T, U2T and U3T in 5 µL were mixed with 5 µL of 2× SB, and then incubated at RT for 15 min. 10 µL of 2× dGLB were then introduced. The resultant mixture was heated to 90°C and cooled at RT for 15 min. 10 µL (out of 20 µL) were taken for dPAGE analysis. Panel e: the boxed DNA bands from panel c were excised and the DNA eluted. The DNA was dissolved in 10 µL of water. 5 µL was subjected to the same procedure as described for panel d. +M: the reaction mixture contained NaCl and $MgCl_2$; -M: the two salts were omitted.

Experimental details for Figure 2. 5 pmol of U2T in 5 µL were mixed with 5 µL of various 4× folding buffers containing 40 mM HEPES (pH 7.5), and 4× concentrations of metal ions listed for each lane. Then 10 µL of 2× dGLB were introduced and the resultant mixture was heated to 90°C and cooled at RT for 15 min. 10 µL (out of 20 µL) were taken for dPAGE analysis.

Experimental details for Figure 3. 5 pmol of U2T in 5 µL were mixed with 5 µL of 4× FB, and 10 µL of 2× dGLB, then heated at 90°C for 5 min and cooled at RT for the specified time before dPAGE analysis. The Ctr lane was an exception where the reaction mixture was immediately loaded following the heat denaturation step. Each reaction was started at different time points so that all the reactions ended at the same time. For example, the 4 h reaction was started one hour after the start of 5 h reaction. 5 µL of each reaction mixture were used for the dPAGE analysis.

Experimental details for Figure 4. 5 pmol of each of listed U2R mutants in 5 μL were mixed with 5 μL of 4× FB and 10 μL of 2× dGLB, and heated at 90°C for 5 min, and cooled at RT for 15 min. 5 μL were taken for dPAGE analysis.

Experimental details for Figure 5. Panel b: 5 pmol of U2R1 or U2R5 in 5 μL were mixed with 5 μL of 4× FB and 10 μL of 2× dGLB, heated at 90°C for 5 min, and cooled at RT for 15 min. 5 μL were taken for dPAGE analysis. Panel c: the same procedure for panel b was used, however, the dPAGE was substituted with native PAGE. Panel d: All samples (500 μL each) contained 4 μM U2R1 or U2R5, 20 mM HEPES, pH 7.5, 150 mM NaCl, 5 mM $MgCl_2$, and 7 M urea. Each sample was heated to 90°C for 5 min and incubated at RT for 20 h. 350 μL was placed in a 0.1 cm quartz cuvette. CD studies were carried out using an AVIV model 410 CD spectrometer (Lakewood, NJ, USA) as per the manufacturer's instructions. The samples were scanned from 320 to 220 nm. All shown spectra are the average of 2 individual scans.

Experimental details for Figure S1. The details are described above under "In vitro selection procedures".

Experimental details for Figure S2. The same procedure described for Figure 2 was followed in this experiment.

Experimental details for Figure S3. The same procedure described for panel d of Figure 1 was followed in this experiment.

Experimental details for Figure S4. 200 picomoles of U2T in 200 μL of water were heated to 90°C for 5 min, and divided into two 100 μL control (the "C" sample), and test (the "T" sample). 100 μL of water was added to the control sample and 100 μL of 4xFB was added to the test sample. Both samples were cooled to room temperature for 35 min. Then 200 μL of 0.4% Dimethyl sulfate was added to both, followed by 35-min incubation. (This procedure allows the U2T to be methylated while folded). The DNA was recovered by ethanol precipitation. Each sample was labeled using 20 μCi of [γ-^{32}P]ATP following the previously described procedure, and precipitated with ethanol, and re-suspended in 5 μL water. The test sample was first mixed with 5 μL of 4× FB, and then with 10 μL of 2× dGLB, while the control sample was mixed with 5 μL of water, and then with 10 μL of 2× dGLB. The resultant mixture was heated to 90°C and cooled at RT for 15 min. The test sample was subjected to dPAGE, to exclude any unfolded molecules in the test sample. The control sample was also purified by dPAGE. The DNA recovered from the gel were then treated with 100 μL of 10% piperidine at 90°C for 30 min, dried by a vacuum concentrator. Each sample was then dissolved in 10 μL of 1× dPAGE loading buffer, heated at 90°C for 5 min, and immediately loaded onto a dPAGE gel.

Experimental details for Figure S5. (a) Samples with volumes of 60 μL contained 1 μM U2R1, 10 mM HEPES, pH 7.5, 150 mM NaCl, 5 mM $MgCl_2$, in the presence of absence of 7 M urea. Corresponding negative control mixtures (with no DNA) were also made. The mixtures were heated to 90°C for 5 min and cooled to RT. 1.2 μL of 100 μM Protoporphyrin IX (PPIX) was added to each sample. After 10 minutes of incubation, samples were scanned with CARY Eclipse Fluorescence Spectrophotometer, with the excitation wavelength of 410 nm, and emission between 550 nm – 750 nm was recorded. (b) The UV measurements were run using Cary 300UV/Vis spectrophotometer.

Samples with volumes of 400 μL were prepared to contain 5 μM U2R1, 10 mM HEPES, pH 7.5, 150 mM NaCl, 5 mM $MgCl_2$, in the presence of absence of 7 M urea, along with corresponding blank mixtures (with no DNA). Each sample and blank was heated at 90°C for 5 min and cool to RT, then loaded into quartz cuvettes. The cuvettes were then loaded into the spectrophotometer. The block temperature was set to 23°C and the samples were incubated for 15 min before the melting experiment was initiated. Absorbance at 295 nm was measured between 23°C to 95°C. The ramp was set at 1°C/min. Melting points were calculated using the derivative of absorbance vs. temperature graphs. (c) 5 pmol of U2R1 or RNA-U2R1 in 5 μL were mixed with 5 μL of 4× FB and 10 μL of 2× dGLB, heated at 90°C for 5 min, and cooled at RT for 15 min. 5 μL was taken for dPAGE analysis. +M: the reaction mixture contained NaCl and $MgCl_2$; -M: the two salts were omitted. NC: 2× dGLB was mixed with 10 μL of RNA-U2R1 dissolved in water.

SUPPLEMENTARY TABLES AND FIGURES

Table S1. DNA sequences isolated after 16 rounds of in vitro selection.

Sequence ID	Nucleotides in random-sequence region[a]	Sequence class
SQ01	GGAGGGGGTGGTGGGTTGTATACTTGGGTGGTTGGGGGGT	UD1
SQ02	AGAGGGGGTGGTGGGTTGTATACTTGGGTGGTTGGGGGGT	
SQ03	GTAGGGGGTGGTGGGTTGTATACTTGGGTGGTTGGGGGGT	
SQ04	GTAGGGGGTGGTGGGTTGTATACTTGGGTGGTTGGGGGGT	
SQ05	GTAGGGGGTGGTGGGTTGTATACTTGGGTGGTTGGGGGGT	
SQ06	GTAGGGGGTGGTGGGTTGTATACTTGGGTGGTTGGGGGGT	
SQ07	GGAGGTGGTGGTGGGTTGTATACTTGGGTGGTTGGGGGGT	
SQ08	GGAGGTGGTGGTGGGTTGTATACTTGGGTGGTTGGGGGGT	
SQ09	GGAGGGGGTGGTGGGCTGTATACTTGGGTGGTTGGGGGGT	
SQ10	GGAGGGGGTGGTGGGCTGTATACTTGGGTGGTTGGGGGGT	
SQ11	GGAGGGGGTGGTGGGTCGTATACTTGGGTGGTTGGGGGGT	
SQ12	GGAGGGGGTGGTGGGTCGTATACTTGGGTGGTTGGGGGGT	
SQ13	GGAGGGGGTGGTGGGTCGTATACTTGGGTGGTTGGGGGGT	
SQ14	GGAGGGGGTGGTGGGTTGCATACTTGGGTGGTTGGGGGGT	
SQ15	GGAGGGGGTGGTGGGTTGTGTACTTGGGTGGTTGGGGGGT	
SQ16	GGAGGGGGTGGTGGGTTGTATGCTTGGGTGGTTGGGGGGT	
SQ17	GGAGGGGGTGGTGGGTTGTATACTCGGGTGGTTGGGGGGT	
SQ18	GGAGGGGGTGGTGGGTTGTATACTTGGGCGGTTGGGGGGT	
SQ19	GGAGGGGGTGGTGGGTTGTATACTTGGGTGGTTGGGGGGG	
SQ20	GTAGGTGGTGGTGGGTTGTATACTTGGGTGGTTGGGGGGT	
SQ21	GTAGGGGGTGGTGGGTCGTATACTTGGGTGGTTGGGGGGT	
SQ22	GTAGGGGGTGGTGGGTTGCATACTTGGGTGGTTGGGGGGT	
SQ23	GGAGGGGGTGGTGGGTTGTATACTTGGGTGGTTGGGGGTC	
SQ24	CGGGGGGTGGCGGGGAAAGAGAGATGGGTGGTCGGGGGGT	UD2
SQ25	CGGGGGGTGGTGGGGGAAGAGAGATGGGTGGTCGGGGGGT	
SQ26	CGGGGGGTGGCGGGTGAAGAGAGATGGGTGGTCGGGGGGT	
SQ27	CGGGGGGTGGCGGGAGAAGAGAGATGGGTGGTCGGGGGGT	
SQ28	CGGGGGGTGGCGGGAGAAGAGAGATGGGTGGTCGGGGGGT	
SQ29	CGGGGGGTGGTGGGAGAAGAGAGATGGGTGGTCGGGGGGT	
SQ30	GGGGGGATGGTGGGGTGGAGAAGTGGGTGGCCGGGGGGT	UD3
SQ31	GGGGGGATGGTGGGGTGGAGAAGTGGGTGGCCGGGGGGT	
SQ32	GTGGGGATGGTGGGGTGGAGAAGTGGGTGGCCGGGGGGT	
SQ33	GGGGGGATGGTGGGGTGGAGAAGTGGGTGGCCGGGGGTC	

[a]: Nucleotide highlighted in yellow denotes a mutation in comparison to the first sequence listed in each class at the same position.

a

**S1:**	5′-ACTCTTCCTA GCFRQGGTTC GATCAAGA
**DL1:**	5′-GCTGACTGCC AAGACNNNNN NNNNNNNNNN NNNNNNNNNN NNNNNNNNNN NNNNNTCTGG TATGTGCTCA
**FP1:**	5′-GCTGACTGCC AAGAC
**RP1:**	5′-TGAGCACATA CCAGA
**RP2:**	5′-AAAAAAAAAA AAAAAAAAAA LTGAGCACAT ACCAGA
**T1:**	5′-GTCTTGGCAG TCAGCTCTTG ATCGA

b

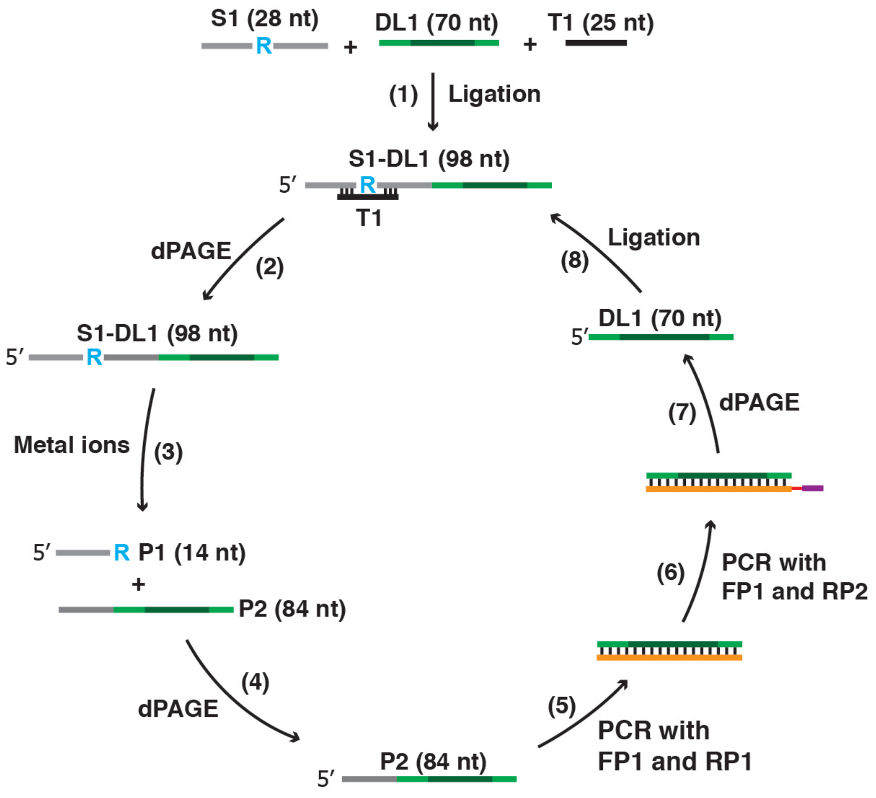

Figure S1. Information for In Vitro Selection. (a) The sequences of the DNA molecules used for the in vitro selection experiment. DNA molecules are color-coded to assist the understanding of panel b. **(b)** In vitro selection scheme with the following 8 steps. (1) Ligation of phosphorylated DL1 (DNA library) to S1 (fluorogenic substrate) templated by T1. (2) Purification of ligated S1-DL1 by dPAGE. (3) Incubation of S1-DL1 in the selection buffer (40 mM HEPES, pH 7.5, 100 mM NaCl, and 10 mM $MgCl_2$). (4) Purification of cleaved products by dPAGE. (5) PCR using FP1 and RP1 as primers. (6) PCR with FP1 and RP2 as primers. Note: RP2 contains a triethylene glycol spacer (L, panel a) and A20 tail at the 5' end; the spacer prevents the poly-A tail from being copied, making the non-DNAzyme-coding strand 20 nucleotides longer than the coding strand. (7) Purification of DL1 strand by dPAGE. (8) Phosphorylating DL1 and ligating it to S1. The cycle of steps 2-10 was repeated for 16 times in this study. The experimental details are described in Section A above under “In vitro selection procedures”.

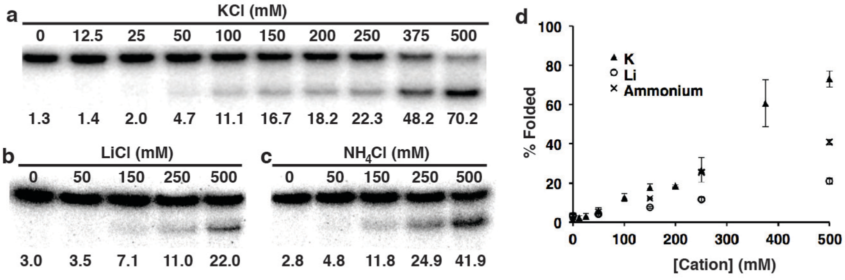

Figure S2. Effect of varying concentrations of K^+, Li^+ and NH_4^+ on the folding of U2T. Each reaction mixture contained 10 mM HEPES (pH 7.5), 7 M urea, and specified concentrations of KCl **(a)**, LiCl **(b)** or NH_4Cl **(c)**. **(d)** % Folded structure vs. cation concentration. The mixture was heated at 90°C for 5 min and left standing at room temperature for 15 min prior to 7MU-dPAGE analysis.

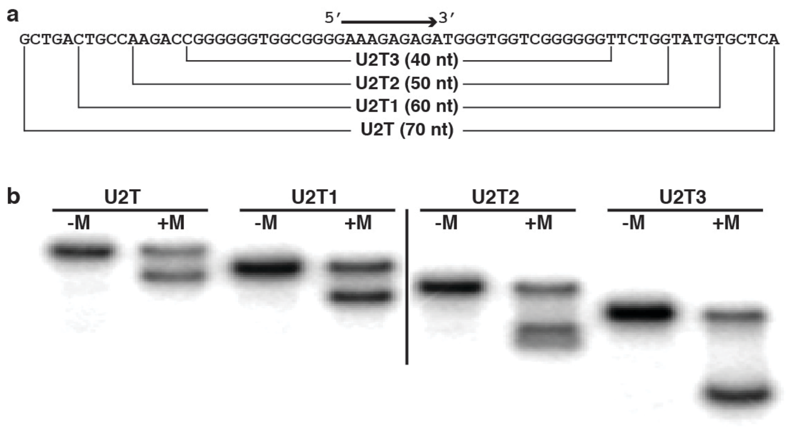

Figure S3. Examination of sequence truncation on the structure folding of UD2. (a) Four progressively shortened UD2 sequences tested for this study. U2T3 is subsequently named U2R as it contains only 40 nucleotides located in the random-sequence region. (b) dPAGE analysis of each truncated sequence in the presence of 10 mM HEPES, pH 7.5, 150 mM NaCl and 5 mM $MgCl_2$ (+M) or 10 mM HEPES only (-M).

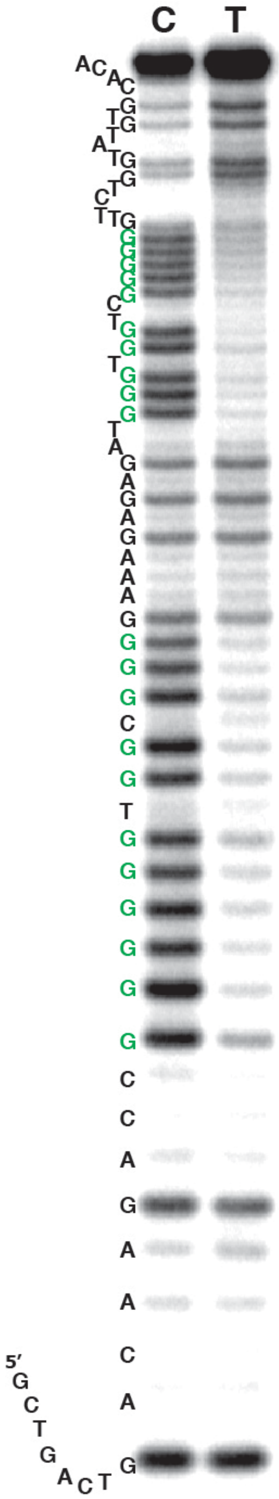

Figure S4. Methylation interference of guanine nucleotides in U2T. C: Control where every G residue can be freely methylated by DMS (dimethyl sulphate). T: Interference Test where the G residues that are important to the folding would show significantly reduced methylation. The guanine residues with significantly reduced band intensity in the T lane in comparison to the same DNA band in the C lane are indicated in green.

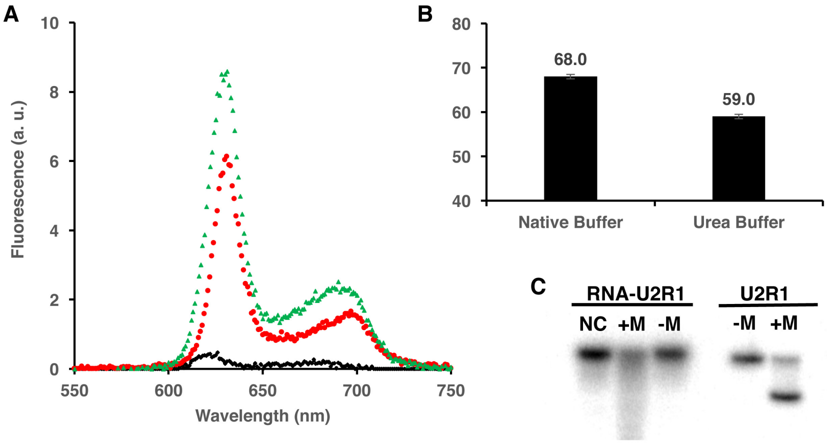

Figure S5. (a) Testing the presence of parallel G-quadruplex in the structure of U2R1 with protoporphyrin IX (PPIX). PPIX is known to produce enhanced fluorescence upon binding to parallel G-quadruplex. Three conditions were tested: PPIX alone (data in black), PPIX with U2R1 (data in red) and PPIX with U2R1 in the presence of 7M urea. (b) UV-melting points of U2R1 obtained under native and urea-containing conditions. (c) dPAGE analysis of RNA-U2R1 in the presence of 10 mM HEPES, pH 7.5, 150 mM NaCl and 5 mM $MgCl_2$ (+M) or 10 mM HEPES only (-M).

Original gel images in Figure 1

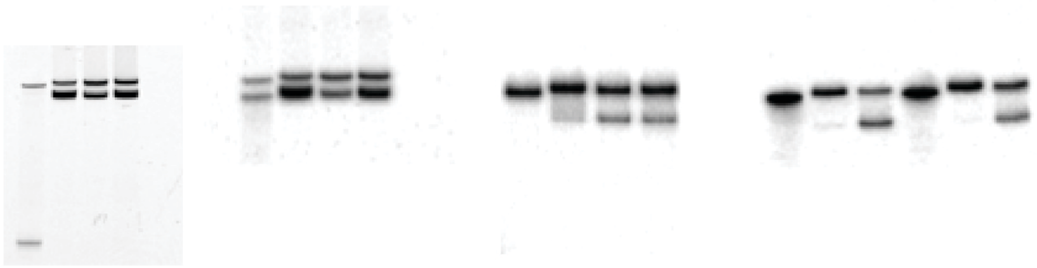

Original gel images in Figure 2

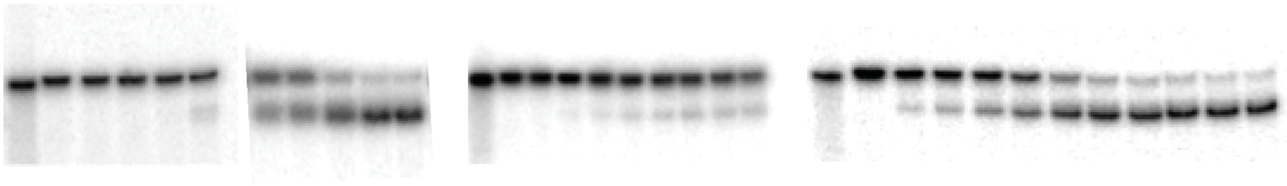

Original gel images in Figure 3

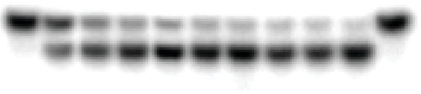

Original gel images in Figure 4

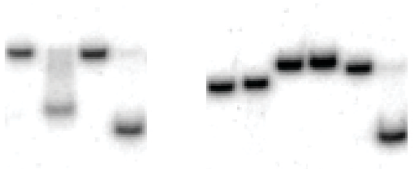

Original gel images in Figure 5

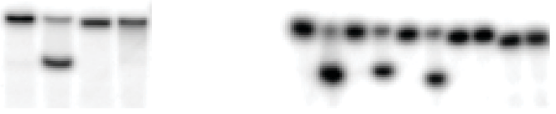

Figure S6. Uncropped gel images for Figures 1-5 of the main manuscript.
